# Lasting alteration of spatial orientation induced by passive motion in rabbits and its possible relevance to mal de débarquement syndrome

**DOI:** 10.3389/fneur.2023.1110298

**Published:** 2023-02-22

**Authors:** Jun Maruta

**Affiliations:** ^1^Department of Neurology, Icahn School of Medicine at Mount Sinai, New York, NY, United States; ^2^Department of Rehabilitation and Human Performance, Icahn School of Medicine at Mount Sinai, New York, NY, United States

**Keywords:** animal model, cross-axis stimulation, otolith, perceptual disorder, phantom sensation, semicircular canal, velocity storage, Coriolis force

## Abstract

**Background:**

Mal de débarquement syndrome (MdDS) is a chronic disorder of spatial orientation with a persistent false sensation of self-motion, whose onset typically follows prolonged exposure to passive motion of a transport vehicle. Development of similar but transient after-sensations mimicking the exposed motion and associated postural instability, indicative of central vestibular adaptation, are common. The cause of MdDS is thought to be a subsequent failure to readapt to a stationary environment. However, vestibular plasticity pertinent to this illness has not been studied sufficiently. Because the rabbit's eye movement is sensitive to three-dimensional spatial orientation, characterizing maladaptation of the vestibulo-ocular reflex (VOR) induced in the animal may open an approach to understanding MdDS.

**Methods:**

Three rabbits underwent a series of 2-h conditioning with an unnatural repetitive motion that involved a complex combination of roll, pitch, and yaw movements in a head-based reference frame, consisting of periodic rolling in darkness in a frame of reference that rotated about an earth-vertical axis. Eye movement in three dimensions was sampled during the conditioning stimulus as well as during test stimuli before and up to several days after conditioning.

**Results:**

During roll-while-rotating conditioning, the roll component of the VOR was compensatory to the oscillation about the corresponding axis, but the pitch component was not, initially prominently phase-leading the head pitch motion but subsequently becoming patently phase-delayed. Unidirectional yaw nystagmus, weak but directionally compensatory to the earth-vertical axis rotation, was seen throughout the period of conditioning. After conditioning, simple side-to-side rolling induced an abnormal yaw ocular drift in the direction that opposed the nystagmus seen during conditioning, indicating a maladaptive change in spatial orientation. The impact of conditioning appeared to be partially retained even after 1 week and could be partially reversed or cumulated depending on the rotation direction in the subsequent conditioning.

**Conclusion:**

The observed reversible long-term maladaptation of spatial orientation as well as the depth of knowledge available in relation to the vestibular cerebellar circuits in this species support the potential utility of a rabbit model in MdDS research.

## Introduction

Spatial orientation signifies readiness for spatial interactions. Mal de débarquement syndrome (MdDS) is an under-recognized and little-understood chronic disorder of spatial orientation characterized by a persistent false sensation of self-motion, which typically onsets following prolonged exposure to passive motion such as being on a cruise ship or airplane (1, 2). A condition known as “sea legs” or mal de débarquement, i.e., a transient after-sensation mimicking the exposed motion, such as rocking, swaying, or bobbing, and associated postural instability lasting for hours to several days, is common and suggests the existence of some form of entrainment in the central vestibular system (3–7). The exact motion stimulus that triggers the condition or why it persists into a chronic form only in some people is not clear. MdDS is considered rare, but its actual prevalence has not been determined (8, 9). The primary manifestation of MdDS is in the continuous false perception of self-motion, or non-spinning vertigo, which lacks objective measures (2, 10). The disorder is debilitating as false motion sensations are accompanied by postural instability and other, likely secondary, physical, cognitive, and affective problems (11, 12).

The key to beginning to address the pathogenesis of MdDS may be found in a maladapted state of the vestibulo-ocular reflex (VOR). In a ground-breaking study, macaque monkeys were conditioned with 2 h of a motion stimulus known as roll while rotating (RWR), after which the monkeys' VOR to simple side-to-side head rolling became abnormal, featuring extraplanar eye movements consisting of unidirectional horizontal nystagmus and vertical nystagmus that alternated the direction with head tilts (13). The vertical nystagmus paralleled that found in human subjects who spent 64 h in a rotating room (14) and was later shown to mimic anomalous eye movements seen in some patients with MdDS (10).

The vestibulo-ocular reflex is malleable to meet imposed changes in the requirement for gaze stabilization due to eyeglasses, injury, illness, environment, or other causes (15–18). Cross-axial angular motion, such as RWR and as might be experienced by a vehicle passenger, causes the inner ear motion sensors, namely the semicircular canals and the otoliths, to signal mismatched movements (19), and thus, repeated exposure can set a stage for central adaptation to the condition, i.e., a new normal. Based on the experimental data from macaque monkeys, it was hypothesized that the velocity storage mechanism of the central vestibular circuitry would acquire an inappropriate sense of vertical (pitch) motion in association with roll motion when conditioned with RWR, and furthermore that similar maladaptation might underlie MdDS (13). Velocity storage is a working memory-like mechanism of self-motion and spatial orientation that supports brainstem ocular and postural reflexes but is also thought to contribute to the perception of self-motion in the cerebral cortex (20–24). Thus, it follows that dysfunction of velocity storage may cause an incorrect perception of self-motion.

The Dai method of MdDS treatment was developed on the premise that, if velocity storage could be maladapted to cause MdDS, re-stimulating its adaptive capacity to correct it could reverse the illness (10, 25). The validity of the Dai method is supported by its broad effectiveness that is often permanent (10, 12, 26–31). MdDS symptoms can be re-triggered in initially successfully treated patients by subsequent exposure to prolonged motion (12), which further indicates that the treatment effect is not due to simple vestibular habituation. However, despite the apparent relevance of RWR-induced VOR maladaptation in monkeys to MdDS in humans, the RWR-induced mistuning of spatial orientation has thus far been characterized only qualitatively, and there is still a wide knowledge gap between transient mal de débarquement and chronic MdDS. Therefore, the conditioning effect of RWR merits further investigation with an animal model with different sensitivity (32).

In human vision, activities as ordinary as reading a book, recognizing faces, or judging facial expressions depend on bringing the selective image on the specialized high-acuity retinal area called foveola that extends only about 1.25° in visual angle (33–35). Consequently, humans continuously execute a range of both voluntary and involuntary eye movements that support scanning, examination, and pursuit of necessarily selective visual objects (36). Even without vision, as in darkness, humans, and in fact many animals, continue to engage in pseudo-visual navigation and exploration (37–41). Such oculomotor behavior may obscure subtle eye movements associated with an anomaly in the central vestibular mechanism, even if present.

In contrast, rabbits' oculomotor behavior is highly exceptional in that the primary goal of their visual system appears to maintain a stable visuospatial environment rather than to fixate on a selective object. The eyes of rabbits are laterally placed and equipped with a horizontally elongated region of retinal ganglion cell concentration, known as the visual streak, rather than the fovea in frontal-eyed species (42). With their unusually large binocular field of vision that nearly covers a complete sphere, rabbits maintain visuospatial stability by orienting the visual streak to the horizon with robust counter-rotation of the eyes against head roll or pitch (42–45). The presence of target pursuit eye movements has not been demonstrated in the rabbit (42), and spontaneous gaze changes are rare when the head is fixed relative to the body (46, 47). It has been stated that rabbit eye movement is rather a postural phenomenon (48). The proof-of-concept findings here support that eye movements in rabbits provide a unique window to the internal representation of spatial orientation as well as its adaptability, and thus to MdDS.

## Methods

### Animal preparation

Three adult female Dutch-belted rabbits weighing approximately 2.2 kg were used in the study. All experimental procedures followed a protocol approved by the Institutional Animal Care and Use Committee of Icahn School of Medicine at Mount Sinai.

**Figure 1:**
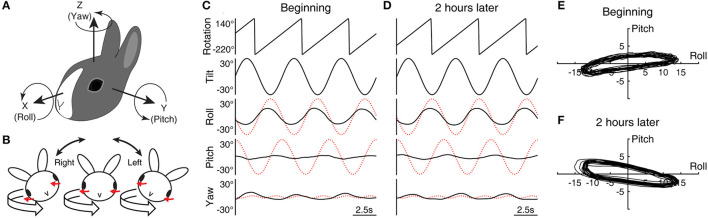
Head and eye movement during conditioning with roll while rotating. **(A)** The head-based coordinate frame used for describing eye movement. **(B)** Cross-axial pitch motion. In the head-fixed reference frame, a rightward or leftward roll tilt respectively evokes a positively- or negatively-directed pitch motion in exchange for yaw motion when combined with leftward rotation (red arrows indicate corresponding compensatory motion). **(C, D)** Sample data (Rabbit 645) of three cycles of rolling at the beginning **(C)** and end **(D)** of the initial 2-h conditioning stimulus with leftward rotation. The eye movement traces have been desaccaded and drift-corrected to mask the effects of nystagmus. The dotted red traces indicate idealized compensatory responses to the stimulus. **(E, F)** Eye position modulation shown in **(C)** and **(D)** re-plotted in the X–Y plane with additional 17 cycles (20 cycles in total).

Using anesthesia, ketamine (32 mg/kg), acepromazine (0.32 mg/kg), and xylazine (5 mg/kg), and under sterile surgical conditions, a head-holding base was implanted on the skull with small screws and dental cement to painlessly immobilize the head during experiments. In the same surgery, two search coils were implanted to record three-dimensional eye movement in a two-field magnetic system. One coil was wound around the limbus of the left eye under the conjunctiva to measure the yaw and roll components of eye movement expressed in head-based coordinates (Figure 1A). The other coil, pre-formed, was inserted under the superior oblique and superior rectus muscles of the same eye and sutured to the globe to measure the pitch component (torsional relative to the orbit) (45). The animals were given at least 1 week before initial testing to recover from the surgery.

### Vestibular stimulus

All experiments were conducted in darkness with the animal's body held in a close-fitting container and the head centered in a multi-axis vestibular stimulator (Contraves Goerz, Neurokinetics, Pittsburg, PA) (49). Rabbits were conditioned with 2 h of RWR in darkness (Figure 1B), consisting of ±30°, 5 s/cycle periodic rolling about an earth-horizontal axis and left- or rightward rotation about an earth-vertical axis at 72°/s (5 s per revolution). The earth-vertical rotation was initiated first, and after the cessation of the per-rotatory nystagmus, the roll component was combined with the ongoing rotation. After 2 h of the combined stimulus, the rolling and then the rotation were stopped, and the decay of post-rotatory nystagmus and a sign of small underdamping, if any, were observed for about 40 s. To test the effect of conditioning, the animals were also given off-vertical axis rotation (OVAR) and sinusoidal tilts about an earth-horizontal axis.

**Figure 2:**
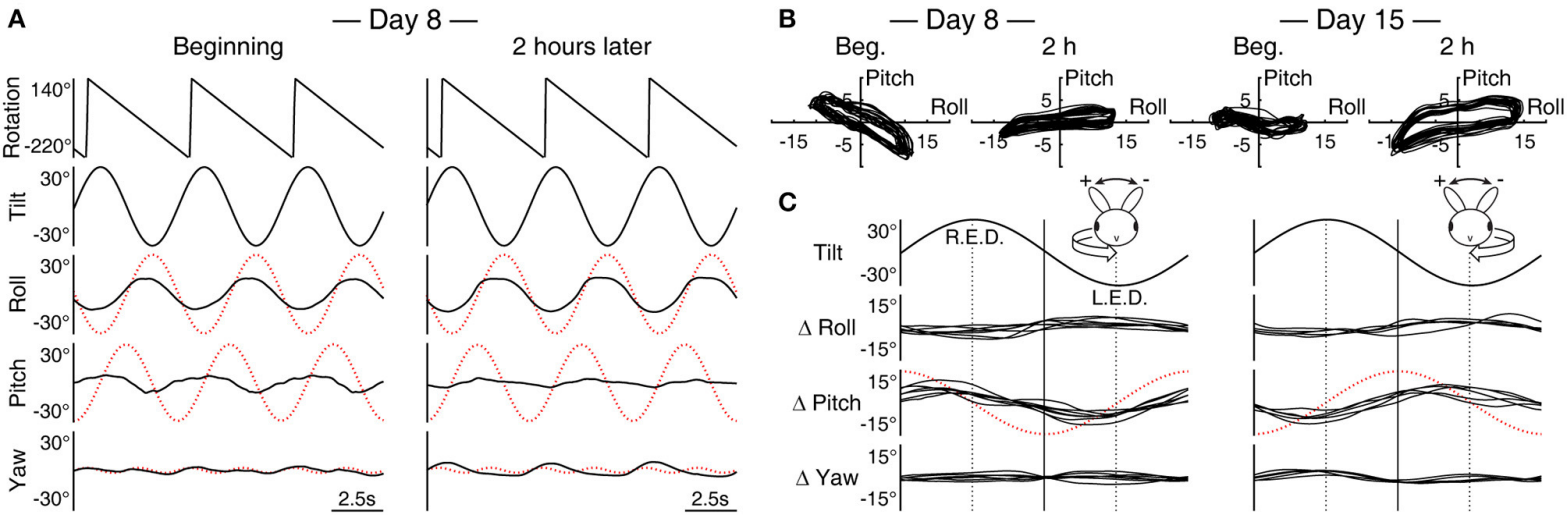
Pitch response during roll while rotating is subject to adaptation. **(A)** Sample data (Rabbit 645) of three cycles of rolling at the beginning and end of the 2-h conditioning stimulus with rightward rotation (c.f., Figures 1C, D, leftward rotation). The dotted red traces indicate idealized compensatory responses to the stimulus. Note that at the beginning, the roll and pitch responses are nearly out of phase with each other, effectively canceling the respectively associated yaw version and vergence to reveal the expected double-frequency modulation in yaw. With a subsequent change in the phase of the pitch response, the yaw modulation takes on a half-wave rectified form. **(B)** The superimposed trajectory of eye position modulation over 20 cycles in the X–Y plane at the beginning and end of the conditioning on Days 8 and 15, both with rightward rotation (Rabbit 645, replotting of **(A)** for Day 8). **(C)** A composite of average differences (Δ) in roll, pitch, and yaw modulations between the beginning and end of the 2-h stimulus with leftward (left column) and rightward (right column) rotation across animals and conditioning trials. R.E.D., right ear down; L.E.D., left ear down. The relation between roll tilt and Δ pitch is inverted between left- and rightward conditioning. Relative to idealized pitch responses (shown with red traces show as in **(A)** but with one half of the amplitude), both left- and rightward conditioning effectively induced large phase lags in pitch responses; however, a corresponding effect was not observed in the pitch responses to simple sinusoidal pitching.

In the head-fixed reference frame, RWR entailed cross-axial pitch angular motion in addition to the applied roll and yaw motion. The head position, α, in the head-fixed roll plane as a function of time, *t*, can be expressed as α = *A*·sin(ω*t*), where *A* now is 30° or π/6 and ω the angular frequency is 2π/5. The idealized compensatory roll eye position modulation with a gain of 1 is −30° × sin(ω*t*) by inverting the phase of this expression (Figures 1C, D, 2A, dotted red traces). The angular velocity in the head-fixed pitch plane is a projection of that of the spatial vertical rotation onto this plane, scaled by sin(α) = sin{*A*·sin(ω*t*)} ≈ sin(*A*)·sin(ω*t*) for a small amplitude of *A*, which has a normalized root-mean-square error of <0.05 for A up to 59.3°. Given sin{π/6·sin(ω*t*)} ≈ 0.5·sin(ω*t*), the angular velocity in the head pitch plane is thus ≈36°/s × sin(ω*t*) for leftward rotation and ≈-36°/s × sin(ω*t*) for rightward rotation. Integrating this result and inverting the phase, the idealized compensatory pitch eye position modulation with a gain of 1 is ≈±29° × cos(ω*t*). Note the approximate symmetry between the idealized roll and pitch responses. Lastly, the angular velocity generated in the head-fixed yaw plane is scaled by cos(α) = cos{*A*·sin(ω*t*)}, which modulates at twice the frequency with a peak-to-peak amplitude of 1 – cos(*A*) around an offset of ≈{cos(*A*) + 1}/2. At steady state, the semicircular canals respond only to the modulatory component, which here are ≈4.8°/s × cos(2 ω*t*) for leftward rotation and ≈-4.8°/s × cos(2 ω*t*) for rightward rotation, from which the idealized compensatory yaw eye position modulation is found to be ≈∓1.9° × sin(2 ω*t*).

Of note, although pitch while rotating (PWR) and RWR are often referred to as Coriolis stimuli in the literature, the Coriolis force may be ruled out as the primary mechanism of consequences since opposing eccentric displacements of the head from the tilt axis by several centimeters do not change eye movement response characteristics (50). Here, the rabbit's head was carefully centered but had a similar displacement been introduced, the amplitude of the extraneous linear acceleration along either the X- or Y-axis of the head (Coriolis and centripetal/translational, respectively) would still have been under 0.01 g (51).

All three rabbits were initially conditioned with rolling and leftward rotation. The animals were tested with additional vestibular stimuli immediately before and after, and up to 12 days after this initial conditioning. The animals were then reconditioned with the rotation direction reversed to the right. The first animal (Rabbit 567) was conditioned only once in each direction of rotation, leftward on Day 1 and rightward on Day 12. The subsequent two animals (Rabbits 645 and 646) were conditioned a total of five times on a weekly basis following an identical schedule: rolling with leftward rotation on Days 1, 22, and 29 and with rightward rotation on Days 8 and 15 (L–R–R–L–L). When not engaged in experiments, the animals were free to move around in their home cage in the animal facility.

In Rabbit 567, the effect of conditioning was tested with OVAR with a 15° and 30° tilt with a 20 s period (constant 18°/s) and sinusoidal tilts about earth-horizontal roll, pitch, and intermediate axes with 3, 5, 7, and 10 s periods with a fixed peak angular speed of 17°/s, which was approximately half of the peak angular speeds of the roll and pitch components of the conditioning stimulus. During steady state, the VOR during OVAR is driven exclusively by the otoliths (52, 53). On the other hand, the VOR during sinusoidal tilts is driven by the coactivation of the vertical semicircular canals and the otoliths. With the different oscillation periods, the positional modulation during sinusoidal tilts varied from ±8° to ±27° so that the otolith contribution to the VOR relative to the semicircular canals presumably increased with longer periods of oscillation. Rabbits 645 and 646 were tested with OVAR with a 15° tilt and 16 s period (constant 22.5°/s) and sinusoidal tilts about earth-horizontal roll, pitch, and intermediate axes with 4, 8, and 16 s periods with a fixed positional modulation amplitude of ±15°, resulting in 23.6, 11.8, and 5.9°/s peak angular speeds, respectively, which presumably varied the contribution of the semicircular canals to the VOR relative to that of the otoliths. For all animals, several cycles of these test stimuli were given twice within each testing session. The order of presentation of sinusoidal tilts with various combinations of orientations and periods was counterbalanced to curtail possible bias.

### Data acquisition and analysis

The voltage outputs of the search coils were calibrated by rotating the magnetic field coils by approximately ±10° about the X, Y, and Z axes independently around the rabbit that was held stationary in space with the head fixed at the center of the field coil frame, during which the animal made no spontaneous eye movement. The rabbit was then fixed to the field coil and the innermost axis of the vestibular stimulator with the head centered. A computer controlled the vestibular stimulator and recorded stimulus and eye movement data that were digitized at 1,000 Hz. Data were sampled for the test stimuli as well as for several minutes at the beginning and end of each trial of RWR conditioning. The data were stored for offline analysis.

Calibrated eye position data were differentiated to yield velocity, and fast phases of nystagmus were identified in the velocity data and replaced with lines connecting the ends. Eye velocity bias was estimated with the offset term of a sine fit. Desaccaded eye velocity data were integrated, and a Fourier filter below the frequency of interest was applied to these re-integrated data. The desaccaded and drift-corrected eye position signals were then fit with a sine function to determine the amplitude of modulation and the phase relation to the stimulus. Sine-fit parameters were also used to characterize the trajectory of eye position modulation in the X–Y (roll-pitch) plane. To assess the effect of RWR conditioning, eye position signals at the initial and terminal 100-s periods were, respectively, superimposed cycle by cycle and averaged. Roll and pitch eye movement in response to X- or Y-axis linear acceleration during OVAR was examined by symmetrically averaging cycle-by-cycle data from left- and rightward rotations to counterbalance the hysteresis effects. The gain and phase of roll and pitch modulations relative to the idealized response as well as yaw drift velocity during sinusoidal tilts were calculated from a sine fit of desaccaded eye velocity data and were vectorally averaged within each test condition.

## Results

### Initial conditioning with leftward rotation

The yaw eye movement during RWR conditioning was composed of cyclic modulation superimposed on weak nystagmus that continued throughout the 2-h conditioning period, with fast phases occurring just once or twice per the 5-s cycle of rolling. Although the slow phase velocity of the nystagmus, estimated as the bias in the cyclic eye velocity modulation, was no more than 1.6°/s in any animal, the nystagmus was always directionally compensatory to the rotation by having leftward fast phases. As for cyclic modulation, the desaccaded and drift-corrected yaw eye position signals often resembled a half-wave rectified form as shown for Rabbit 645 as an illustrative example (Figures 1C, D, bottom), which was likely a combination of the expected double-frequency modulation (dotted red traces) and vergence and version eye movements that are known to, respectively, accompany head/eye pitch and roll (44).

Rabbit 645's initial roll response had a gain of 0.44 and a phase lead of 15.9° relative to the idealized response, while the pitch response had a much smaller gain of 0.08 and a much larger phase lead of 60.1° relative to the idealized response (Figure 1C). Two hours later, the roll response was nearly unchanged with a gain of 0.45 and phase lead of 13.3°, but the pitch response adopted a gain of 0.11 and phase *lag* of 30.3° (Figure 1D). While the idealized response would have drawn a nearly circular trajectory of eye position modulation in the X–Y plane, the actual trajectory was substantially flattened at both time points (Figures 1E, F). The large phase change in pitch during conditioning was reflected in the positive-to-negative change in the long-axis slope of the flattened trajectory (+7.3° to −9.3°).

Rabbit 567's initial and terminal roll responses had a gain of 0.27 and 0.41 and a phase lead of 24.8° and 21.5°, respectively, relative to the idealized response. The pitch responses during the respective periods had a gain of 0.08 and 0.15 and a phase *lead* of 91.6° and a *lag* of 34.4° relative to the idealized response. Thus, the pitch phase again underwent a pronounced change during the stimulus, from a strikingly large initial lead relative to the idealized response to a substantial lag. The long-axis slope of the eye position modulation trajectory in the X–Y plane reduced from 17.5° to −17.1°. Rabbit 646's corresponding roll responses had a gain of 0.24 and 0.41 and phase lead of 12.9° and 7.4°, respectively, and the pitch responses had a gain of 0.21 and 0.14 and phase lead of 56.5° and 18.3°, respectively. Although the change in the pitch phase in Rabbit 646 was less pronounced compared to the other animals, the long-axis slope in the X–Y plane nevertheless reduced from 34.3° to 3.9°.

### Subsequent conditioning

All three rabbits were subsequently re-conditioned with rolling and rightward rotation. As with the initial conditioning, weak but directionally compensatory yaw nystagmus, this time with rightward fast phases, was found throughout the conditioning period. The reversal of the rotation direction also inverted the relation of the cross-axial pitch kinematics to the roll tilts (Figures 1C, D vs. Figure 2A). With this inversion, the pitch response presented with a large phase lead relative to the idealized response initially, which again changed to a substantial lag during 2 h of conditioning. In comparison, the roll response remained more stable. Correspondingly, the long-axis slope of the eye position modulation trajectory in the X–Y plane turned from negative (567: −19.8°; 645: −28.5°; 646: −35.8°) to positive (567: 3.8°; 645: 6.8°; 646: 14.6°) (Figure 2B, Day 8, shown for 645).

Rabbits 645 and 646 were further re-conditioned with rolling and rightward rotation 1 week later. The long-axis slope of the eye position modulation in the X–Y plane was initially negative but by a lesser extent than the beginning of the previous conditioning (645: −7.1°; 646: −2.3°), which after 2 h became positive once again (645: 12.8°; 646: 10.8°) (Figure 2B, Day 15, shown for 645). On Days 22 and 29, these animals were re-conditioned with rolling and leftward rotation as on Day 1. The long-axis slopes in the X-Y plane for Rabbits 645 and 646, respectively, were Day 22 beginning, 10.4° and 3.9°, end, −35.8° and −23.7; and Day 29 beginning, −17.0° and −10.9, end, −39.4 and −35.8. Thus, a pattern that emerged in the slope changes associated with reversing the direction of the conditioning rotation and reapplying it (L–R–R–L–L) was an initial large effect that was partially retained after 1 week, which in turn was subject to a further, cumulative change.

Figure 2C shows *changes* in the roll, pitch, and yaw components of cycle-by-cycle superimposed average eye position signals across animals and trials derived as a start-to-finish difference (Δ) during each conditioning trial. The changes in pitch, albeit with varied amplitudes between ±3.5° and ±10.3° (mean ±6.2°), were polarized in relation to the direction of the conditioning rotation, nearly in or out of phase with respect roll tilt for left- and rightward conditioning, respectively. In contrast, in any change in roll or yaw, specificity with the direction of the conditioning rotation was not evident. The roll response, however, often increased the modulation amplitude during conditioning regardless of the direction of rotation, with changes up to ±5° (mean ±2.9°) being always nearly out of phase with roll tilts.

### Off-vertical axis rotation

**Figure 3:**
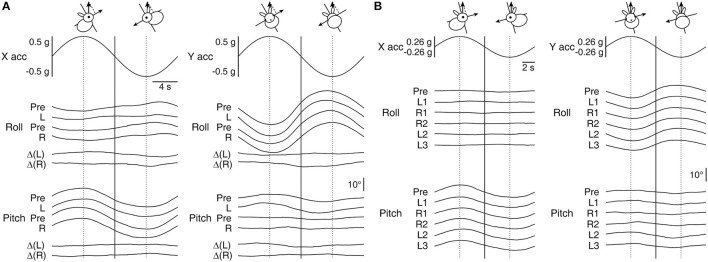
Roll and pitch eye movement in relation to the modulation of linear acceleration along the X- and Y-axes during OVAR. **(A)** Averaged responses by Rabbit 567 to ±18°/s rotation about an axis tilted by 30° immediately before (Pre) and after the left- and rightward conditioning on Days 1 (L) and 12 (R), respectively. The bottom two traces in each panel depict the corresponding before-after differences (Δ). **(B)** Averaged responses by Rabbit 646 to ±22.5°/s rotation about an axis tilted by 15° immediately before (Pre) and after the conditioning on Day 1 (L1) and the subsequent conditioning on Days 8 (R1), 15 (R2), 22 (L2), and 29 (L3). Vertical lines are drawn to visualize phase relations among the traces and the head orientations that resulted in maximal linear acceleration projected along the X- or Y-axis by gravity.

That the above-found changes in pitch responses during RWR conditioning might be expressed afterward as an alteration in the otolith-mediated VOR was tested with OVAR. When the otolith-ocular reflex is induced with tilts, the response is known to be approximately proportional to the projection of gravity on the head-fixed X–Y plane, rather than to the tilt angle (44, 54–56). Accordingly, Figure 3 illustrates roll and pitch eye position modulations in relation to the modulation of linear acceleration along the X- and Y-axes expressed in g.

**Figure 4:**
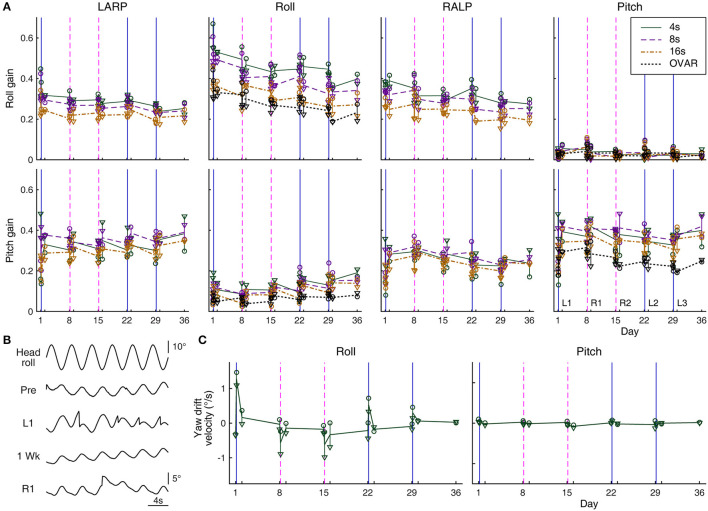
Eye movement response to sinusoidal tilts. **(A)** Roll and pitch gains during ±15° oscillations about differently oriented axes measured over days: −45° relative to roll (in the plane of the left anterior and right posterior canals, LARP); roll; 45° relative to roll (in the plane of the right anterior and left posterior canals, RALP); and pitch. Circles and triangles represent the responses of Rabbits 645 and 646, respectively. Solid green, dashed purple, and dash-dot brown lines represent average responses to 4-, 8-, and 16-s oscillations, respectively. In roll gain, these lines are generally seen to be arranged from top to bottom in respective order, but in pitch gain, the arrangement is more mixed. Responses to OVAR with a 15° tilt and 16-s period are also shown with dotted black lines for comparison (computed only for the roll and pitch axes as in Figure 3B). Solid blue and dashed magenta vertical lines indicate the timing of left- and rightward conditioning, respectively. **(B)** Sample raw yaw eye position data during 4-s period head rolling (Rabbit 645) immediately before (Pre) and after the conditioning on Day 1 (L1) and immediately before (1 Week) and after the conditioning on Day 8 (R1). In L1 and R1, saccades are seen to reset a unidirectional drift. Note the instability in the drifting direction in Pre, which is common in experimentally naïve rabbits. **(C)** Yaw drift velocities measured over days during 4-s period roll and pitch oscillations (Rabbits 645 and 646). The graphical notations are as in **(A)**.

The main eye movement outcomes of X- and Y-axis linear accelerations were in pitch and roll, respectively, in which appreciable post-conditioning changes were not detected. As expected, there were also extraplanar roll and pitch responses to X- and Y-axis linear accelerations, respectively (43, 44), but a post-conditioning emergence of a new response in these regards was not evident either. Specifically, despite that both the conditioning RWR and OVAR with a 30° tilt, as applied to Rabbit 567, assumed equivalent Y-axis accelerations through a periodic alternation between 30° right- and left-ear down tilt positions, and that the animal's pitch response during RWR resulted in considerable reshaping, the pitch response to the Y-axis component of linear acceleration during OVAR was nearly identical before and after each conditioning trial (Figure 3A). The numerical changes in pitch eye modulation amplitude to Y-axis acceleration associated with the first and second conditioning trials were only ±0.9° [Δ(*L*)] and ±0.4° [Δ(*R*)], respectively, which hardly accounted for those of ±5° and ±3.8° during conditioning in the same animal (Figure 2C). This animal's response to OVAR with a 15° tilt was smaller but qualitatively similar and again without a clear indication of a change. The series of conditioning in Rabbits 645 and 646 also did not reveal a clear systematic change associated with conditioning in the response to OVAR (Figure 3B, shown for Rabbit 646), except that the roll response decreased over the course of weeks of experimentation (Figure 4A, top row, second panel from left, dotted black lines).

### Sinusoidal tilts

The possible post-conditioning alteration was also looked for in the VOR during the coactivation of the vertical semicircular canals and the otoliths in the form of sinusoidal tilts. Across rabbits, the roll eye movement to head rolling phase-lead the ideal compensation by ≈10° and the pitch eye movement to head pitching by ≈13° without any clear dependence on the period or amplitude of the oscillation or the temporal relation to the conditioning stimulus. These phase values were comparable to those previously reported from the same laboratory using a similar experimental setup (44).

The response gains in the roll were more variable than the phases. For Rabbit 567, which underwent sinusoidal tilts with a fixed peak angular speed of 17°/s with varying periods, the pre-conditioning roll gain to head rolling was ≈0.54 (range 0.47–0.58) without any clear dependence on oscillation periods, having as much variability within any given period as among the different periods. However, the roll gain just after either left- or rightward conditioning tended to be larger (range 0.52–0.86), more so for oscillations with shorter periods, during which the relative contribution of the otoliths was presumably smaller. For Rabbits 645 and 646, which underwent sinusoidal tilts with a fixed positional modulation amplitude of ±15° with varying periods, the roll gain was generally lower for oscillations with longer periods, during which the relative contribution of the semicircular canals was presumably smaller (Figure 4A, top row, second panel from left). Transient post-conditioning roll gain changes in these rabbits, if any, were not consistent with those in Rabbit 567 or between themselves, and thus may reflect experimental variability. However, in both Rabbits 645 and 646, the roll gains decreased over the weeks of experimentation. This decrease paralleled that in the overall smaller roll response during OVAR, to which the semicircular canals had no contribution (Figure 4A, same panel, dotted black lines).

To head pitching with a fixed peak angular speed of 17°/s, Rabbit 567 responded with a gain of ≈0.31 (range 0.24–0.41) without showing any clear dependence on oscillation periods just before or just after either left- or rightward conditioning. Essentially comparable gains were obtained for Rabbits 645 and 646 in response to ±15° pitching with varying periods (Figure 4A, bottom right). These pitch responses were larger than those to OVAR, both of which appeared stable over the weeks of experimentation. Overall, the pitch response to sinusoidal head pitching did not reflect the alteration in the pitch response that developed during RWR conditioning.

There were always extraplanar roll and pitch eye movements during sinusoidal tilts as the VOR of the rabbit is often appreciably non-coplanar with the stimulus (43, 44). If the extraplanar pitch response to simple head rolling were to be altered in the manner of the pitch response during RWR conditioning or as suggested by experimental results in monkeys and humans (13, 14), the new pitch response component would be in and out of phase with the head rolling after left- and right-ward conditioning, respectively. However, the results were equivocal at best. For Rabbit 567, the numerical changes followed this pattern only for sinusoidal rolling with a 10-s period, and even for this condition, the average amplitudes of change were equivalent to just ±1.7° and ±2.2° per ±30° of head roll in association with left- and right-ward conditioning, respectively, which were smaller than the corresponding changes during RWR conditioning (±6.0° and ±7.0°, respectively). For Rabbits 645 and 646, the numerical changes followed the expected pattern only for sinusoidal rolling with 4- and 8-s periods, and the amplitudes of these changes were again small (range ±0.3°-±3.1° per ±30° of head roll). The pitch or roll responses to sinusoidal tilts about the intermediate axes also did not reveal a pattern associable with the conditioning stimuli (Figure 4A, first and third columns from left).

Paralleling the post-RWR nystagmic response in yaw to simple sinusoidal head rolling in macaque monkeys (13), the rabbits responded to simple head rolling with a yaw positional drift, a leftward drift following leftward conditioning and a rightward drift following rightward conditioning. Unlike in monkeys, however, the yaw drift was small, barely developing into a nystagmic form (Figure 4B). For Rabbits 645 and 646, the drift was most prominent during the fastest rolling (4-s period, 23.6°/s peak angular speed) and essentially absent during the slowest (16-s period, 5.9°/s peak angular speed), whereas for Rabbit 567, the drift was less affected by the oscillation periods between 3 and 10 s (all 17°/s peak angular speed) but less prominent overall.

Despite the subtle presentation of these drifts, the impact of a conditioning regimen was still apparent when tested 1 day and 1 week later. The pattern shown in Figure 4C, left panel, indicated the conditioning impact diminishing over days to a slight bias, which then could be cumulated or reversed depending on the direction of rotation in the subsequent conditioning. Note the directionally systematic nature of the induced yaw drift, which differed from eye position instability during sinusoidal oscillation common in experimentally naïve rabbits (57), as also observed in present experiments (Figure 4B, “Pre”).

No yaw drift was induced with sinusoidal head pitching with any period at any time (Figure 4C, right panel). Oscillations about an intermediate axis, approximately in the plane of the left anterior and right posterior semicircular canals, often induced a yaw drift resembling that during roll oscillations following leftward, but not rightward, conditioning. Conversely, oscillations approximately in the plane of the right anterior and left posterior semicircular canals often induced a yaw drift resembling that during roll oscillations following rightward, but not leftward, conditioning. Therefore, RWR conditioning asymmetrically altered the central interpretation of semicircular canal-otolith coactivation in the vertical canal planes.

## Discussion

Head tilts during rotation are unnatural and known to be severely perceptually disorienting (14, 58, 59). Nevertheless, the brain is equipped with the ability to reconstruct the sense of the ongoing rotation during such a stimulus, even in darkness without vision, as indicated by the production of directionally compensatory nystagmus during PWR, RWR, or similar stimuli (19, 50, 60). How the brain carries out such computations is presently unknown. However, after plugging the bilateral horizontal semicircular canals, yaw nystagmus can still be induced during PWR/RWR but not after plugging the four vertical canals or after the extinction of velocity storage with bilateral cuts of horizontal canal afferent nerves (19, 50). Furthermore, with only a single push-pull pair of vertical canals from the two sides intact and all the other canals plugged, tilting in the plane of the intact pair during rotation does not induce compensatory yaw nystagmus while tilting in the orthogonal plane (“null plane”) does so maximally (19). In the latter, the intact vertical canal pair is activated as a result of the cross-axial, “fictitious” angular acceleration generated in the rotating frame of reference, to which the otoliths are insensitive. These demonstrations indicate that the reconstruction of the sense of rotation, a remarkable feat of spatial orientation, takes place by way of resolving spatio-temporal incongruency between the otolith- and vertical canal-driven signals. Specifically, in the case of RWR, such incongruency occurs between the otolith-driven roll signals and the vertical canal-driven pitch signals.

A key question at hand is how the brain adapts to RWR during a long exposure in darkness, presumably while confronted with the unverifiable constant presence of rotation. To this end, rolling in an environment where the sense of rotation is visually evoked with optokinetic stimulation can alter the VOR in a like manner to RWR in darkness but in the opposite polarity (13); thus, during RWR in the light, where the presence of rotation is visually supported, the opposing effects may significantly blunt the adaptive drive. During RWR, the cross-axial pitch angular kinematics are aptly signaled by vertical semicircular canal afferents (19). However, the present results indicate that the pitch response during RWR in darkness is not the direct reflection of the vectoral sum of these afferent activities as might be expected for the role of the VOR to compensate for the detected head motion (61) but a highly synthetic outcome of a central mechanism. It is not clear whether this pitch response serves a useful orienting, as opposed to compensatory, function that can facilitate early visual processing or is merely a curious byproduct of a normal neural computation under the unnatural stimulus. Regardless, results indicate that the synthesis of the pitch response undergoes a drastic modification during extended exposure to RWR in darkness. The utility of this modification is also unclear, but such flexibility suggests that this central machinery continuously monitors and attempts to resolve sensory conflicts, a function associated with velocity storage (23, 58, 62). Results also indicate that, once the machinery adapts to the continuous presence of motion within a time frame of hours, the maladapted state can be retained for a significantly longer term. That is, spontaneous or active recovery from such maladaptation is slow or limited, signifying the unusual nature of the conditioning stimulus, presumably lacking counteracting stimuli in normal sensorimotor interactions. Moreover, the conditioning effect may be cumulated or partially reversed depending on the composition of the subsequent RWR. An apparent exception was the cumulated attenuation of ocular counter-rolling, which was noted regardless of the direction of the rotation that combined with the head rolling, and might have been contributed by repetitive rolling by itself.

In the post-RWR state, the rabbits' VOR to simple head rolling was found with a yaw drift, driven expressly in the direction that opposed the unidirectional yaw nystagmus during the RWR conditioning. A parallel, although much stronger, response was previously found in RWR-conditioned macaque monkeys (13). The present examination hinted that the strength of the anomalous yaw eye drift in rabbits might depend on the amplitude of the head rolling velocity, suggesting a contribution of the vertical semicircular canals in the drift generation. Thus, the computational mechanism that decodes rotation during RWR is plausibly also responsible for the post-conditioning yaw velocity generation during simple rolling, possibly by falsely detecting incongruency between the otolith- and vertical canal-driven signals in such a way that suggests the presence of reversed rotation.

This interpretation is in line with the hypothesis that the velocity storage mechanism acquires an inappropriate sense of pitch motion in association with roll motion when conditioned with RWR (13). Since this hypothesis maintains pitch motion to be interpreted still as it is after RWR conditioning, the idea also appropriately represents the present finding in the rabbits that there was no clear alteration in the roll, pitch, or yaw component of the VOR response to simple head pitching, despite that the pitch eye movement during RWR conditioning was robustly altered. Such formulation suggests that RWR conditioning distorts motion representation in the roll-pitch plane, although the remapping may be non-linear; the asymmetry that was found in the yaw drift generation during oscillations about the intermediate axes may require a complex explanation.

The hypothesis about the acquisition of a sense of pitch motion with roll motion was originally formed on the basis that the post-RWR VOR during simple head rolling in macaque monkeys prominently featured pitch nystagmus that modulated with the head rolling (13). However, presently in the rabbits, a parallel post-conditioning alteration in the extraplanar pitch response during head rolling was not indicated. The discrepancy may be reconciled if the pitch response in the monkeys were the outcome of the phenomenon known as cross-coupling, signifying a gravity-modulated effect that generates a re-oriented outcome from a drive for yaw nystagmus, yielding a pitch component during a lateral tilt (62–64). This interpretation, when considering the up-down asymmetry that exists in yaw-to-pitch cross-coupling, might also be able to address the phase relationship among roll, pitch, and yaw velocity modulation during post-RWR head rolling in the monkeys (13, 64). In the rabbits, the weak yaw drift may not have translated into a detectable pitch modulation.

As examined with eye position modulation during OVAR, the acquisition of a sense of pitch tilt in association with roll tilt was also not evident in the exclusively otolith-mediated VOR after RWR conditioning. Eye position responses to OVAR resemble those to static tilts and are evidently non-reflective of velocity storage activation (44, 65). Thus, the presumed RWR-induced distortion in the central representation of the roll-pitch plane appears to manifest in eye movement only within special circumstances of interactions between otolith- and semicircular canal-generated signals. The enigmatic nature of such vestibular maladaptation is reminiscent of the phantom motion sensation reported by patients with MdDS, which is difficult to characterize in terms of physical signs including eye movement (10). Of note is a possible parallel between roll-induced yaw drift and the utility of the Fukuda stepping test in the clinical MdDS assessment, in which the presence of left or right turning/drifting is examined while the patient takes alternating steps in place with the eyes closed and the arms extended forward (10, 12, 26, 66). The stepping motion may cause subtle roll head movement or possibly stand in for such since velocity storage can be activated by multiple sensory modalities (24, 67–69).

A direct connection between RWR and MdDS has not been elucidated as yet. However, the success of the Dai treatment developed following the RWR experiment in macaque monkeys (10, 12, 13) was as though MdDS suddenly emerged as the picture to be assembled among pieces of knowledge collected in decades-long pursuit of the velocity storage mechanism (25, 70). MdDS was previously considered intractable (8), but the epiphany that velocity storage may be involved in MdDS opened opportunities to target the root cause of the illness under a unified vestibular system-based perspective and bring about positive long-term outcomes. Velocity storage can be activated even without an explicit sense of rotation embedded in the stimulus such as in the case of flash induced nystagmus (71, 72). Premorbid asymmetry in the vestibular or visual sensitivity may also contribute to the activation of velocity storage while moving about in the environment. It is therefore conceivable that an RWR-like condition for vestibular maladaptation can be set up for some individuals on an undulating ship with flickers of bright sunlight reflected on the surrounding ocean waves, for example.

Vestibular plasticity involving the flocculus, the hemispheric part of the vestibulocerebellum, has been under intense investigation for decades (73–76). The three-dimensional oculomotor consequences associated with the floccular microzonal organization, concomitant with the role of such plasticity, have also been characterized (77, 78). On the other hand, plasticity involving its vermal counterpart, the nodulus, is not yet well established. Given the roles of the nodulus and the adjacent uvula in the control of velocity storage (79–81) as well as the evidence of a stored representation of previously exposed motion in nodulo-uvular Purkinje cells (82), there may be an exciting avenue to explore cellular-level plasticity in the nodulo-uvula in relation to MdDS.

The goal of the present study was to demonstrate the feasibility of utilizing a rabbit model in MdDS research, and thus, the data are restricted and necessarily descriptive. Likewise, the discussion outlined above remains tentative. Furthermore, in macaque monkeys, there is an asymmetry between the responses to PWR and RWR, such that PWR induces more intense yaw nystagmus than RWR (13, 19, 50), and that conditioning with PWR does not result in VOR maladaptation mirroring that after RWR (13). Accordingly, roll head movements during activation of velocity storage have been suggested to have a specific role in the generation of MdDS (10, 83). The purported differences between PWR and RWR were not explored in the present study. Despite these limitations, several positive points may be made. Rabbits are amenable to modification of the VOR by prolonged exposure to a repetitive vestibular stimulus as shown here and previously by others (84, 85). Because of the high sensitivity of the rabbit's VOR to spatial orientation and its modifiability, characterizing a maladapted VOR in the animal promises an attractive approach to understanding MdDS. An added advantage is that because of the large size of rabbit eyes, it is relatively easy to record precise three-dimensional eye movement in this animal using a search coil method (44, 45, 86). Three-dimensional video-oculography is also feasible in this animal (87). Foveal vision is likely not a prerequisite for MdDS as suggested by a putative case of the illness triggered by a rough sea trip in a patient with bilateral retinal ablation (88). The rabbit may make a favorable species of choice because of not only the stated physical and behavioral characteristics but also the depth of knowledge available in this species about the vestibular-cerebellar circuits that support spatial orientation (48, 89, 90).

## Conclusion

The sense of balance supports readiness to interact with the environment, essential to which is the input from the vestibular organs in the inner ear that function as motion sensors. Head movement signals generated by the semicircular canals and the otoliths are re-combined in the brain to support, with the aid of vision and proprioception, an overall sense of orientation and balance. The recently emerged association of velocity storage with the pathogenesis of MdDS gives hope that the neural basis of the illness may be understood to advance treatment. While understanding of vestibular computation has thus far been best advanced in single dimensions, balance involves three-dimensional motion, and it would be fruitful to leverage multi-dimensional vestibular paradigms in experimentation.

Maladaptive vulnerability of central vestibular mechanisms that normally support spatial orientation in three dimensions may come in varying forms, and relatedly, the courses of events that lead to MdDS in individual patients must be disparate. The outcomes of MdDS may still be improved regardless of specific etiologies. A key direction in future research may be developing tool sets to identify dysfunction of velocity storage and to induce plasticity to correct such dysfunction based on solid neuroscientific grounding. The reversible long-term maladaptation of spatial orientation presently demonstrated in the oculomotor behavior of rabbits as well as the depth of knowledge available in the species in relation to the vestibular cerebellar circuits support the potential utility of a rabbit model in MdDS research.

## Data availability statement

The datasets presented in this article are not readily available because the raw data were collected using custom-made software. However, the datasets will be made available by the author upon reasonable request. Requests to access the datasets should be directed to JM, jun.maruta@mssm.edu.

## Ethics statement

The animal study was reviewed and approved by the Institutional Animal Care and Use Committee of Icahn School of Medicine at Mount Sinai.

## Author contributions

JM conceived and conducted experiments and drafted the manuscript.
